# Avocado (*Persea americana*) fruit extract (2*R*,4*R*)-1,2,4-trihydroxyheptadec-16-yne inhibits dengue virus replication via upregulation of NF-κB–dependent induction of antiviral interferon responses

**DOI:** 10.1038/s41598-018-36714-4

**Published:** 2019-01-23

**Authors:** Yu-Hsuan Wu, Chin-Kai Tseng, Ho-Cheng Wu, Chih-Ku Wei, Chun-Kuang Lin, Ih-Sheng Chen, Hsun-Shuo Chang, Jin-Ching Lee

**Affiliations:** 1 Institute of Basic Medical Sciences, College of Medicine, National Cheng Kung University, Tainan, Taiwan; 2 Graduate Institute of Medicine, College of Medicine, Kaohsiung Medical University, Kaohsiung, Taiwan; 3 Department of Biotechnology, College of Life Science, Kaohsiung Medical University, Kaohsiung, Taiwan; 4 Doctoral Degree Program in Marine Biotechnology, College of Marine Sciences, National Sun Yat-Sen University, Kaohsiung, Taiwan; 5 School of Pharmacy, College of Pharmacy, Kaohsiung Medical University, Kaohsiung, Taiwan; 6 Graduate Institute of Natural Products, College of Pharmacy, Kaohsiung Medical University, Kaohsiung, Taiwan; 7 Research Center for Natural Products and Drug Development, Kaohsiung Medical University, Kaohsiung, Taiwan; 8 Department of Medical Research, Kaohsiung Medical University Hospital, Kaohsiung, Taiwan

## Abstract

Dengue virus (DENV) caused millions of infections around the world annually. Co-infection with different serotypes of DENV is associated with dengue hemorrhagic shock syndrome, leading to an estimate of 50% death rate. No approved therapies are currently available for the treatment of DENV infection. Hence, novel anti-DENV agents are urgently needed for medical therapy. Here we demonstrated that a natural product (2 *R*,4 *R*)-1,2,4-trihydroxyheptadec-16-yne (THHY), extracted from avocado (*Persea americana*) fruit, can inhibit DENV-2 replication in a concentration-dependent manner and efficiently suppresses replication of all DENV serotypes (1–4). We further reveal that the NF-κB-mediated interferon antiviral response contributes to the inhibitory effect of THHY on DENV replication. Using a DENV-infected ICR suckling mouse model, we found that THHY treatment caused an increased survival rate among mice infected with DENV. Collectively, these findings support THHY as a potential agent to control DENV infection.

## Introduction

Dengue virus (DENV) belongs to the Flavivirus genus in the *Flaviviridae* family^1^. DENV contains an ~11-kb a positive single-stranded genomic RNA which encodes a single polypeptide^2^. The polyprotein is cleaved by viral and host protease to generate mature structural and nonstructural proteins, including C, prM, E, and NS1~NS5 proteins^3^. DENV infects over 390 million people and causes tens of thousands of deaths every year in the tropical and subtropical countries^4^. The symptoms of DENV-infected patients range from classic flu-like dengue fever (DF) to severe life-threatening diseases including dengue hemorrhagic fever (DHF) and dengue shock syndrome (DSS)^5,6^. DHF is characterized by symptoms of plasma leakage, thrombocytopenia, liver enlargement, and hemoconcentration^7^. DSS is the most serious complication of DHF, which occurs when circulatory failure is detected in addition to DHF symptoms^8^. DEVN is divided into 4 serotypes (DENV-1–4), which are considered regarding the clinical manifestations of dengue fever^9^. Cross-infection by different serotypes of DENV increases the risk of DHF and DSS progression^9^. Nowadays, non-FDA-approved medicines are available to cure DENV infection and DENV-related diseases; therefore, development of new therapeutic drugs or supplements against DENV infection is an important issue.

The innate immune responses, especially the type I interferon (IFN-I) pathway, are the important action of early host defense against pathogen^10^. Virus infection is recognized by pattern-recognition receptors (PRRs), which subsequently activate several transcription factors, such as nuclear factor-kappa B (NF-κB)^11–13^. In normal conditions, NF-κB is retained in the cytoplasm in an inactive form by binding to inhibitors of κBα (IκBα). Upon pathogen stimulation, IκB kinase (IKK) activation initiates IκBα phosphorylation and the degradation of IκBα, which leads to the translocation of NF-κB into the nucleus to trigger IFN regulatory factor (IRF) and IFN-I expression and secretion. IFN-I bound to cell surface IFN receptor (IFNAR) phosphorylates Jak1 and 2, and then subsequently phosphorylated transcription factors STAT1 and STAT2^14^. Subsequently, phosphorylated STAT1 and STAT2 form the transcription complex ISGF3 with IRF9^15^ and enter the nucleus to trigger IFN-sensitive response element (ISRE) for the expression of antiviral IFN-stimulated genes (ISGs), including 2′–5′-oligoadenylate synthetase (OAS)1, OAS2, OAS3, and protein kinase R (PKR). Activation of these ISGs leads to the inhibition of virus replication^15–17^. In contrast, antiviral IFN-mediated responses can be hindered by viruses, for example, DENV NS2B/NS3 protease, NS5, and NS4B can block IFN signaling via different mechanisms^15,18,19^. However, increasing reports demonstrate that enhancement of endogenous IFN and downstream antiviral gene expression by compounds or natural products can overcome DENV suppression of IFN responses to effectively inhibit DENV infection *in vitro* and *in vivo*, although the detailed mechanism is not yet clearly identified^20,21^. Therefore, enhancement of antiviral IFN is still considered a potential antiviral strategy against DENV replication.

*Persea americana* Mill. (*P*. *americana*) belongs to the family Lauraceae, which is known as avocado and widely grows in tropical and subtropical regions^22^. The fruit, stem, and leaf of avocado are widely used in ethno-medicine^23^. Particularly, the avocado fruit contains lots of nutrients such as vitamin E, vitamin B, potassium and monosaturated fatty acids^24^, which have been reported to exhibit several bioactive properties such as antibacterial^25^, antiviral^26^, antioxidant^27^, anti-atherosclerotic^22^, hepatoprotective, and other activities^23,27^. The bioactive components of avocado contain monoterpenoids, sesquiterpenoids, triterpenoids, flavonoids, alkaloids, steroids, carotenoids and long-chain fatty alcohol derivatives^27^. In the present study, we performed a drug screen of several compounds isolated from the unripe fruit of avocado^27^, including oleic acid (OA), (2 *R*,4 *R*)-1,2,4-trihydroxyheptadec-16-ene (THHE), (2 *R*,4 *R*)-1,2,4-trihydroxyheptadec-16-yne (THHY), avocadenol A, avocadenol C, and avocadoin, and found that THHY exhibited anti-DENV activity without cytotoxicity. We next characterized that THHY inhibits DENV infection through induction of NF-κB-mediated antiviral IFN responses. Finally, we assessed the potential of THHY as a dietary supplement used for prevention of lethal DENV replication using a DENV-infected ICR suckling mouse model.

## Results and Discussion

### Avocado extract THHY exhibits anti-DENV activity

**Figure 1:**
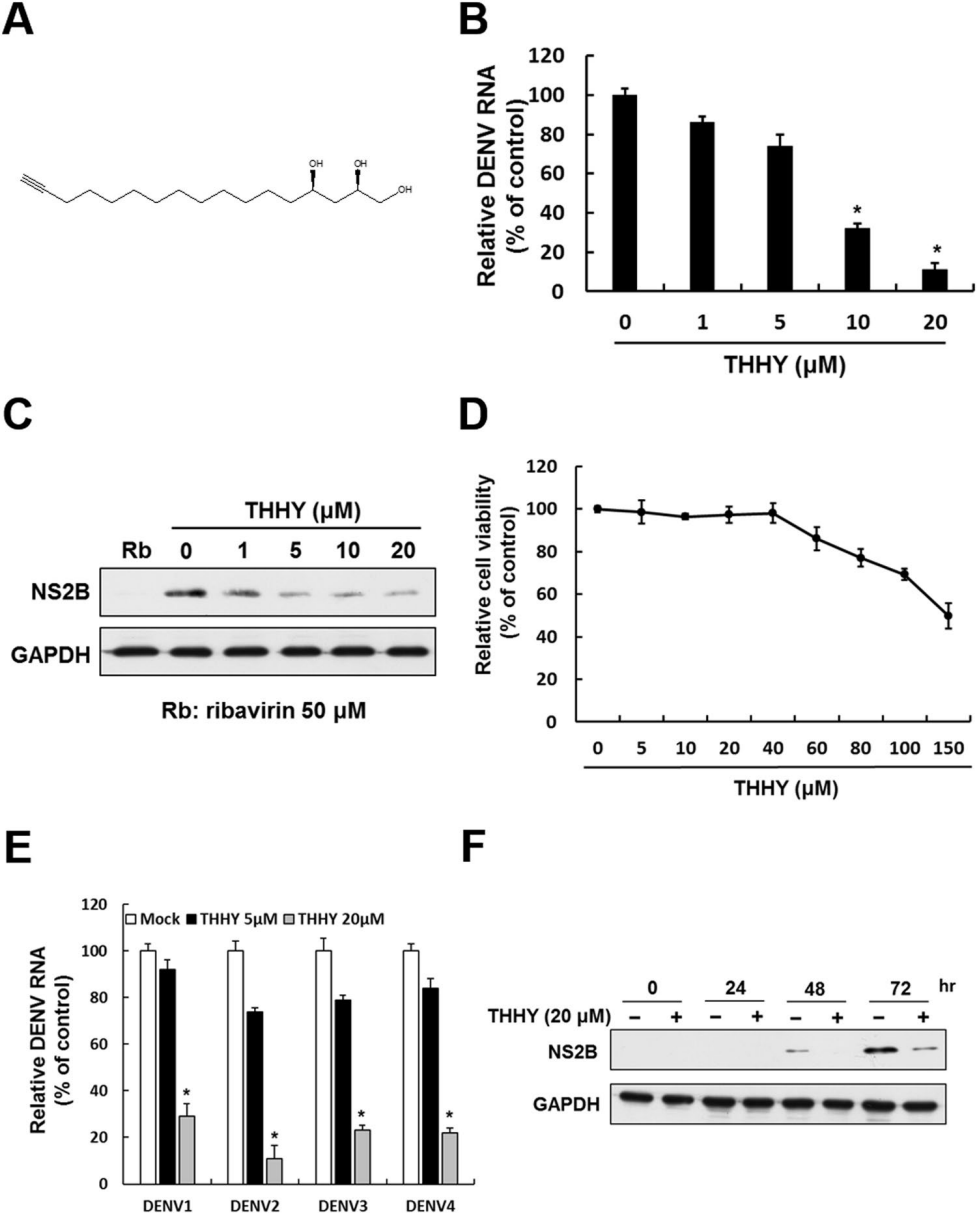
Avocado extract THHY exhibits anti-DENV effect *in vitro*. (**A**) Structure of THHY. (B-C) DENV RNA replication and protein synthesis were inhibited by THHY. DENV-infected Huh-7 cells were incubated with 0, 1, 5, 10 and 20 μM of THHY for 3 days. The treatment of 50 μM ribavirin was served as a positive control. The treatment of 0.1% DMSO, marked “0”, was served as a negative control. Total cellular RNA and protein were harvested for analysis of DENV (**B**) RNA replication and (**C**) protein synthesis levels by qRT-PCR and western blotting, respectively. (**D**) Cell viability of THHY. Huh-7 cells were incubated with indicated concentrations of THHY, and the cell viability was determined by MTS assay after 3 days. (**E**) THHY inhibits replication of 4 serotypes of DENV. Huh-7 cells were infected by 4 serotypes of DENV followed by THHY treatment at concentration of 0 (white bar), 5 (black bar) and 20 (grey bar) μM. Cellular RNA was collected for analysis of RNA levels of DENV at day 3 post-treatment. (**F**) THHY time-dependently inhibits DENV replication. Error bars represent the means ± SD from 3 independent experiments (n = 3). *P < 0.05.

Based on the primary anti-DENV screening of several constituents extracted from avocado using a cell-based DENV infectious system, we identified a component (2 *R*,4 *R*)-1,2,4-trihydroxyheptadec-16-yne (Fig. 1A), named THHY, with significant anti-DENV activity. To further confirm the anti-DENV activity of THHY, DENV-infected Huh-7 was incubated with THHY at doses of 0, 1, 5, 10, and 20 μM. The RNA and protein were harvested to analyze DENV RNA and protein levels at day 3 post-infection, respectively. THHY significantly suppressed DENV-2 RNA and protein synthesis, with EC_50_ values of 10.98 ± 1.9 μM. As expected, the THHY also inhibited the DENV-2 protein synthesis when compared to the non-THHY treated DENV-infected cells, in which the ribavirin was served as a positive control (Fig. 1C). In the meantime, the results of the MTS assay indicated no significant cytotoxicity at the effective concentration (Fig. 1D). Additionally, we further determined the anti-DENV activity of THHY on DENV-1, -2, -3 and -4 (EC_50_ values of 14.61 ± 2.4, 10.98 ± 1.9, 12.87 ± 1.7 and 14.61 ± 2.1 μM) (Fig. 1E). Moreover, we performed a time-dependent antiviral activity assay in Huh-7 cells. The DENV-infected cells were treated with 20 μM of THHY for different period of time. Following 1, 2, and 3 days post-infection, cell lysates were harvested to analyze DENV protein levels by western blotting. The data indicated that THHY inhibited DENV replication in a time-dependent manner (Fig. 1F).

### THHY inhibits DENV replication by NF-κB-mediated IFN production

**Figure 2:**
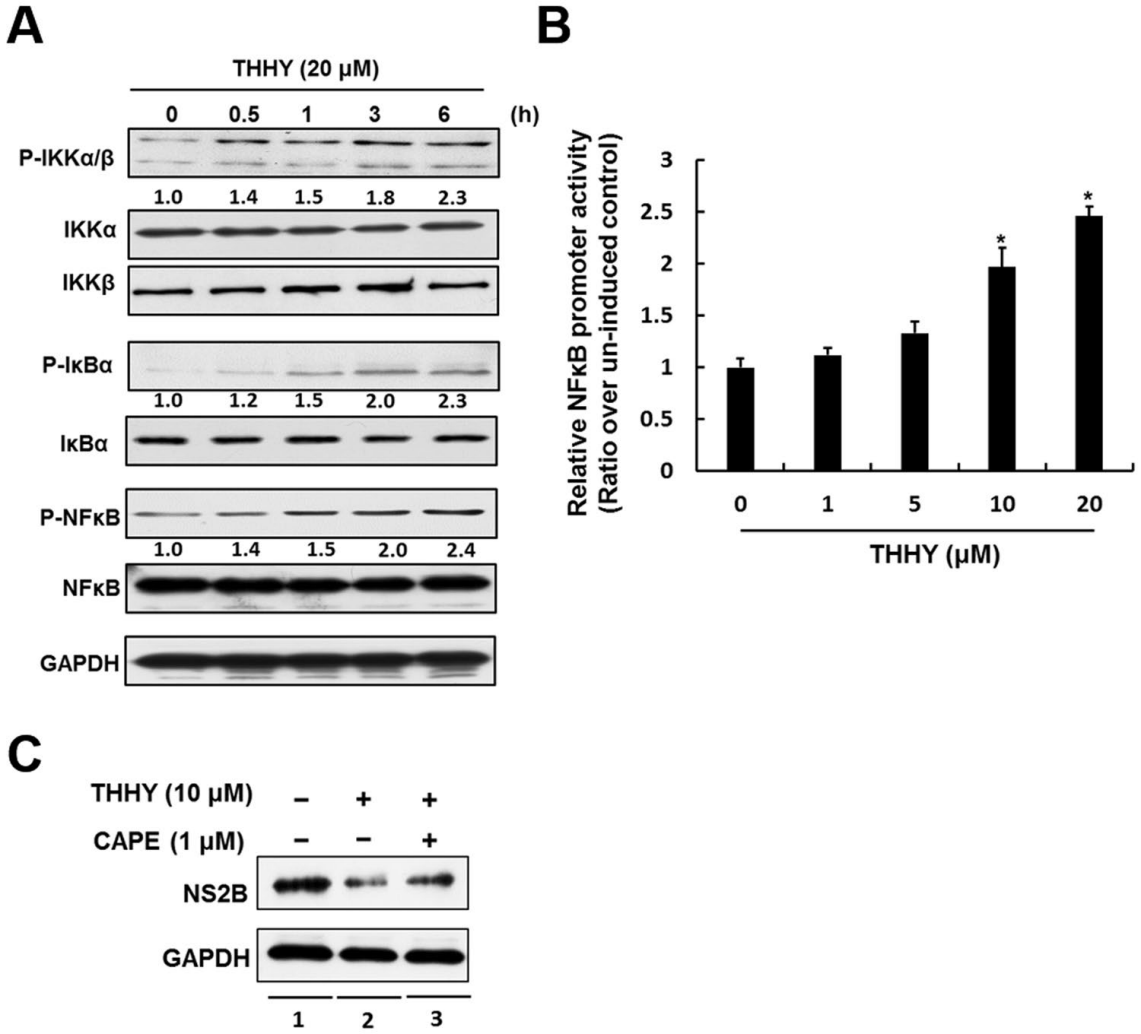
THHY inhibits DENV replication via up-regulation of NF-κB activity. (**A**) THHY induces NF-κB activity upon DENV replication. Huh-7 cells were infected by DENV at an MOI of 0.2 and then treated with 20 μM THHY. Total cellular protein was harvest at 0,0.5,1,3, and 6 h post treatment. NF-κB (P-NFκB), IKK-α/β (P-IKKα/β), and IκBα (P-IκBα) phosphorylation were determined by western blotting with anti-phosphorylated NF-κB, IKK-α/β and IκBα antibodies, respectively. The total NF-κB (NFκB), IKK-α/β (IKKα and IKKβ) and IκBα (IκBα) levels were determined by western blotting with anti-NF-κB, IKK-α/β, and IκBα antibodies, respectively. (**B**) THHY induces NF-κB promoter activity upon DENV replication. Huh-7 cells were transiently expressed pNF-κB-Luc and then infected by DENV at an MOI of 0.2. After virus infection, cells were treated with THHY with indicated concentrations, and the luciferase activity was measured at day 3 post-treatment. (**C**) NF-κB specific inhibitor, CAPE, attenuates the anti-DENV effect of THHY. The DENV-infected Huh-7 cells were co-treated with 20 μM of THHY and 1 μM of CAPE for 3 days. Total cell lysates were harvested for analysis of DENV protein levels by western blotting. The treatment of 0.1% DMSO, marked “0”, was served as a negative control. Error bars represent the means ± SD from 3 independent experiments (n = 3). *P < 0.05.

**Figure 3:**
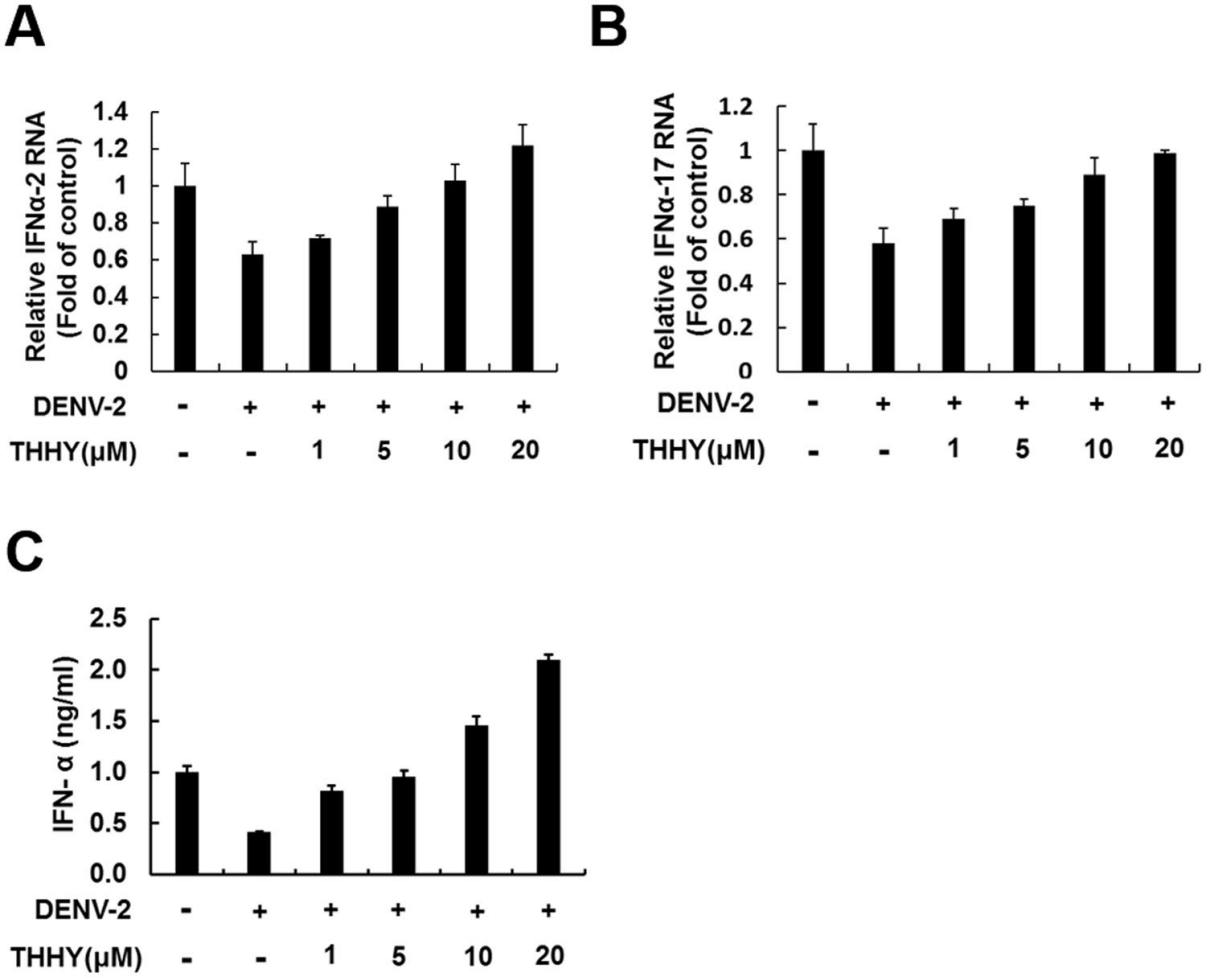
THHY inhibits DENV replication via induction of antiviral IFN production. (**A**–**C**) DENV-infected Huh-7 cells were incubated with THHY at indicated concentrations. Total cellular RNA was collected for analysis of (**A**) IFN-α-2 and (**B**) IFN-α-17 mRNA levels by qRT-PCR at day 3 post-infection. The supernatant was harvested for measurement of (**C**) IFN-α protein level using ELISA kit. The untreated cells served as control and their expression level was set to 1. The non-THHY treated sample was added 0.1% of DMSO as a negative control. Error bars represent the means ± SD from 3 independent experiments (n = 3).

Activation of the nuclear factor-κB (NF-κB) signal pathway is considered as a critical factor for stimulating type I IFN responses against pathogen infection^13^. Recent research demonstrated that induction of NF-κB- mediated antiviral IFN-α responses could efficiently suppress DENV-2 replication^13^. To characterize how THHY inhibits DENV-2 replication, we first analyzed whether THHY treatment could induce NF-κB activity in the presence of DENV. Huh-7 cells were infected by DENV-2 and then incubated with THHY. The total cell lysates were harvested at 0.5, 1, 3, and 6 h after treatment, and the phosphorylation status of NF-κB and its upstream regulators including IκBα and IKKα/β were examined by western blotting. As shown in Fig. 2A, THHY treatment resulted in the accumulation of phospho-IKKα/β and phospho-NF-κB levels in a time-dependent manner. In addition, we performed NF-κB promoter-based reporter assay to identify whether NF-κB transcriptional activity was induced by THHY. As shown in Fig. 2B, THHY dose-dependently induced NF-κB promoter activity upon DENV infection (Fig. 2B). To further confirm the role of NF-κB on anti-DENV activity of THHY, NF-κB specific inhibitor, CAPE, was employed to inhibit NF-κB activity in the presence of DENV infection. As shown in Fig. 2C, THHY effectively reduced DENV protein replication (lanes 1 and 2), and the CAPE treatment attenuated the anti-DENV effect of THHY (lanes 2 and 3). We next examined whether THHY treatment could up-regulate IFN-α levels upon DENV infection. DENV-2-infected Huh-7 cells were incubated with THHY at indicated concentration. The cellular RNA was collected to analyze mRNA levels of FN-α-2 and IFN-α-17 at day 3 post-infection. The results showed that THHY induced both IFN-α RNA level in DENV-2-infected cells (Fig. 3A,B). Furthermore, we simultaneously measured the secretory protein level of IFN-α in the supernatant by ELISA. As expected, IFN-α secretion was increased by THHY treatment (Fig. 3C). Collectively, these results revealed that THHY inhibits DENV replication via up-regulation of NF-κB-mediated antiviral IFN-α expression.

### THHY induces antiviral IFN responses through the STAT pathway

**Figure 4:**
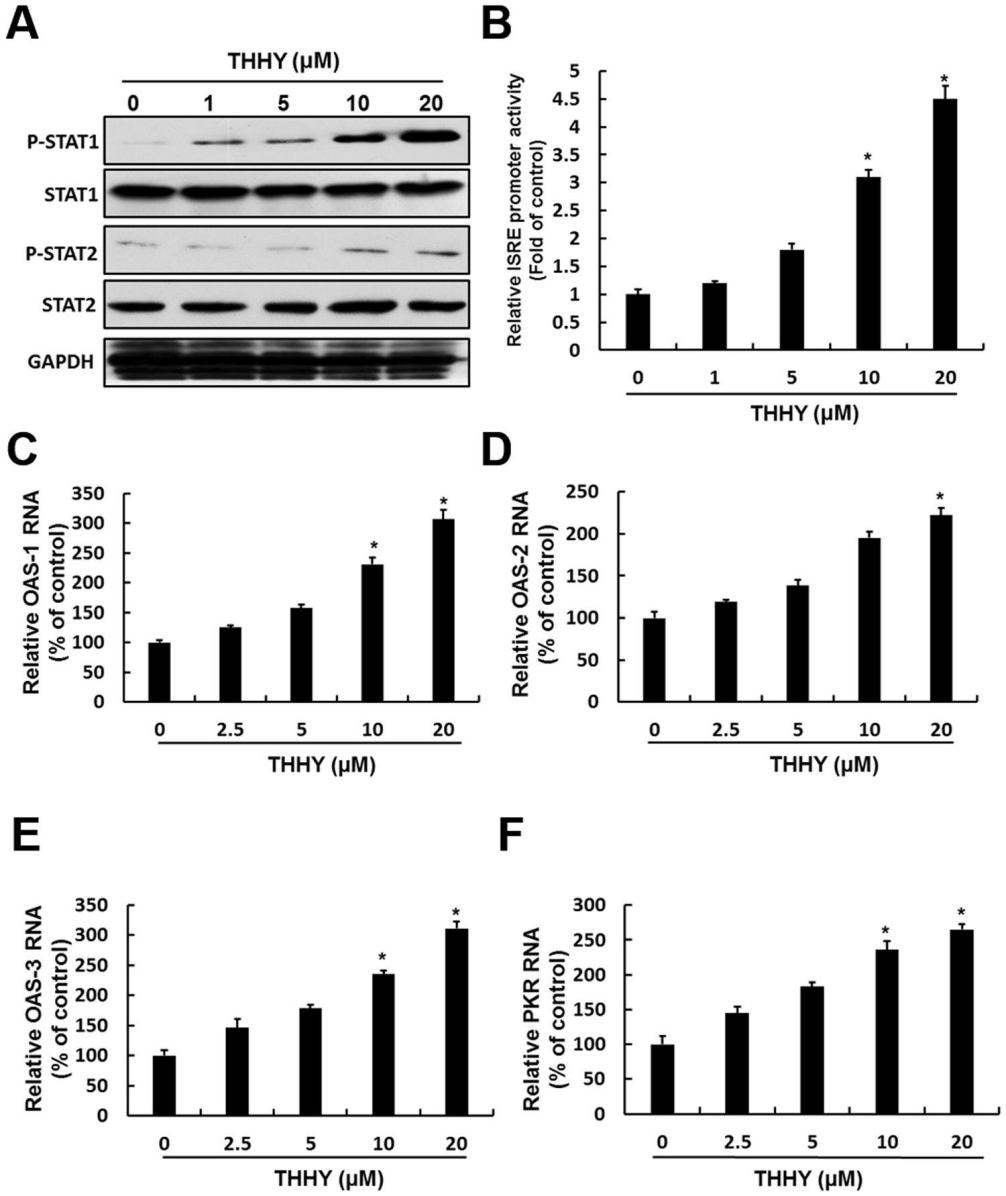
THHY inhibits DENV replication by increasing STAT activity and downstream antiviral gene expression. (**A**) THHY enhances STAT1 and 2 phosphorylation. DENV-infected Huh-7 cells were incubated with THHY at indicated concentrations. Total cellular protein was harvested at day 3 post-infection, and STAT1 and STAT2 phosphorylation levels were determined by western blotting with anti-phosphorylated STAT1 (P-STAT1) and STAT2 (P-STAT2) antibodies, respectively. The total STAT1 (STAT1) and STAT2 level (STAT2) were identified by anti- STAT1 and STAT2 antibodies, respectively. (**B**) THHY enhances ISRE activity. Huh-7 cells were transfected with pISRE-Luc and infected with DENV, then followed by incubation of THHY at indicated concentrations. After 3 days treatment, luciferase activity was analyzed. (**C**–**F**) THHY induces antiviral gene expression. Huh-7 cells were incubated with THHY at indicated concentrations. The treatment of 0.1% DMSO, marked “0”, was served as a negative control. The cellular RNA was harvested to analyze the relative mRNA level of (**C**) OAS1, (**D**) OAS2, (**E**) OAS3 and (**F**) PKR by qRT-PCR. Error bars represent the means ± SD from 3 independent experiments (n = 3). *P < 0.05.

Induction of IFN-α should result in the phosphorylation/homodimerization of STAT1/STAT2 which translocates into nucleus to bind the type I IFN-responsive element (ISRE), ultimately stimulates critical antiviral genes expression including OAS1–3 and PKR^15^. To investigate whether THHY can induce STAT1 and STAT2 phosphorylation in the presence of DENV, the DENV-2-infected Huh-7 cells were incubated with THHY at indicated concentration. After 3 days, total cellular protein was harvested to examine the level of phospho-STAT1 and phospho-STAT2. The results of western blotting showed that THHY treatment increased STAT1 and STAT2 phosphorylation levels in the presence DENV-2 replication (Fig. 4A). Using an ISER-driven luciferase reporter assay, we observed, as expected, that THHY increased ISRE transcriptional activity in a dose-dependent manner (Fig. 4B). Moreover, the results of RT-qPCR revealed that the levels of IFN-stimulated antiviral gene expression were increase by THHY treatment in DENV-2-infected cells (Fig. 4C–F). Collectively, these results support that THHY inhibits DENV-2 replication via upregulation of IFN-α and STAT1/2-triggered antiviral responses.

### THHY protects ICR suckling mice against life-threatening DENV infection

**Figure 5:**
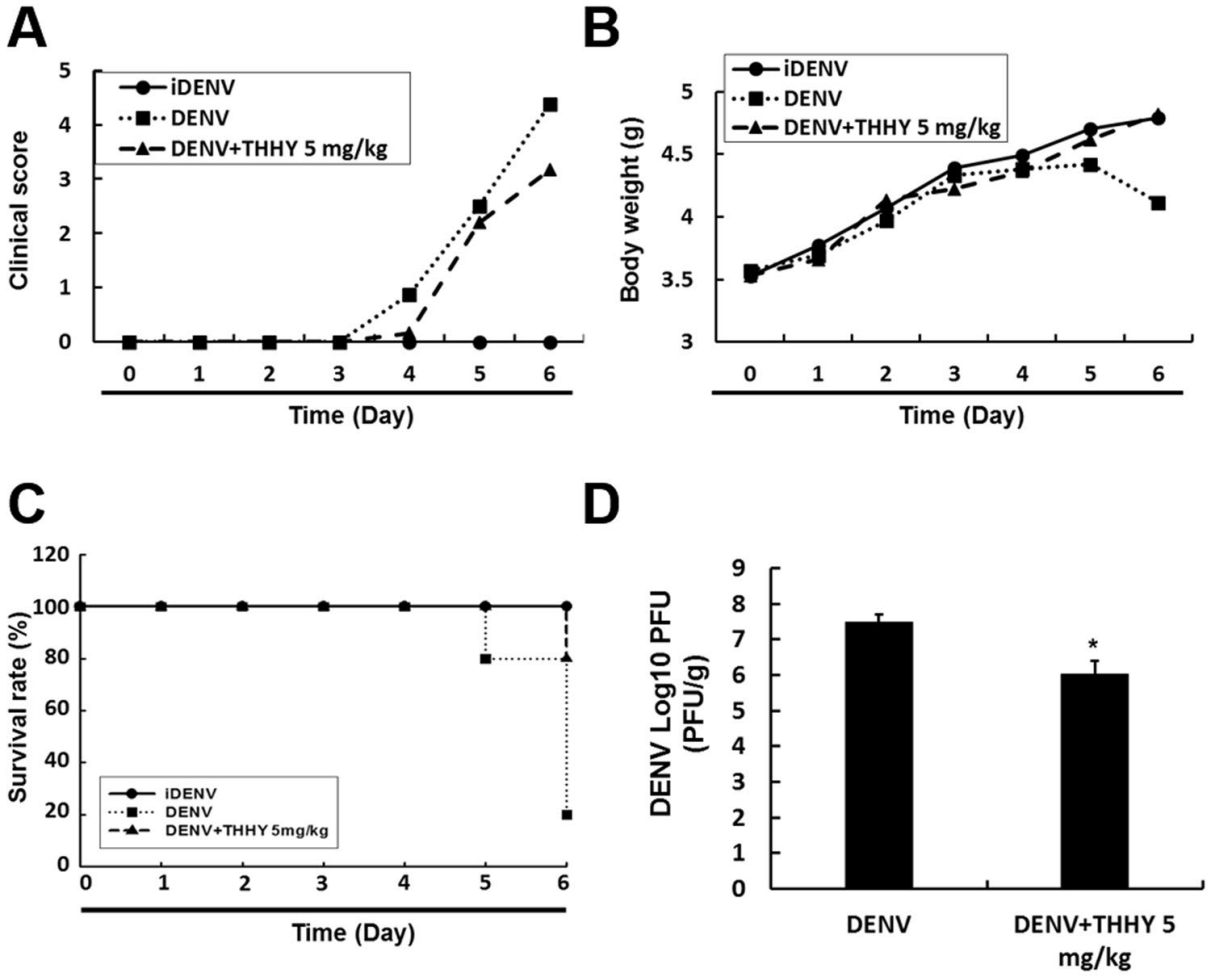
THHY protects ICR suckling mice against lethal DENV infection. (**A**–**D**) Six-day-old ICR suckling mice were i.c. injected with heat-inactivated DENV (iDENV, filled circles, n = 5) or active DENV. DENV-infected mice received saline (DENV, filled squares, n = 5) or 5 mg/kg (DENV + THHY 5 mg/kg, filled triangles, n = 5) THHY inoculation at 1,3,5 days post infection. The (**A**) clinical scores, (**B**) body weight, and (**C**) survival rates were monitored daily until sacrifice at day 6 post-infection. Disease severity was scored as follows: 0: no symptoms, 1: slight weight loss and ruffled hair, 2: slowing of activity, 3: asthenia, 4: paralysis and mortal ill, and 5: death. (**D**) The brain tissue was collected for determination of viral titer by plaque-forming assay. Error bars represent the means ± SD. *P < 0.05.

We further used the ICR suckling mice to evaluate the anti-DENV activity of THHY *in vivo*^20^. Six days old ICR suckling mice were inoculated with active or heat-inactivated DENV-2 (iDENV) by intracerebral injection (i.c. injection), and mice received iDENV served as a negative control. The DENV-2-infected mice were received 5 mg/kg of THHY or saline by intracerebral injection. The clinical scores, body weight and survival rate were recorded every day. After 6 days infection, mice were sacrificed, and the brain tissues were harvested to analyze viral titer using plaque assay. The results showed that the DENV-2-infected mice receiving THHY decreased approximately 40% clinical scores compared to mice receiving saline (Fig. 5A). In addition, THHY treatment recovered about 95% the body weight of infected mice, as compared to iDENV-infected mice receiving saline (Fig. 5B). Notably, THHY increased 60% the survival rate compared with saline treatment in DENV-2-infected mice (Fig. 5C). We further evaluated the decrease in viral titer in DENV-infected brain tissue with THHY treatment (Fig. 5D).

Currently, two direct antiviral agents, NITD-008 and balapiravir, have entered clinical trial phase. However, both trials have been stopped due to toxicity and lack of potency^28–30^. In the present study, we found that THHY, extracted from avocado, exhibits anti-DENV activity *in vitro* and *in vivo* (Figs 1 and 5). Avocado is known to be a healthy fruit which contains many phytochemicals with high antioxidant activity^27^. An association between increased oxidative stress and disease severity of DENV-induced pathogenesis has been reported^31–33^. In addition to blockage of DENV replication, the avocado extracts will facilitate research into the nutritional food additives used in reducing the risk of DENV-induced DHS/DSS in DENV-infected patients. In the study of inhibitory properties against DENV of THHY, we clearly verified that THHY suppressed DENV replication through up-regulation of NF-κB-mediated antiviral IFN responses (Figs 2–4), which is consistent with previous reports that stimulation of IFN-induced antiviral pathway is a promising strategy against DENV infection^20,21^. In the antiviral IFN signaling pathway, the RIG-I-mediated MAVS is a major signaling pathway activating the NF-κB pathway and its downstream antiviral IFN responses^18^. In addition, activation of RIG-I triggers IRF3 and IRF7 expression to induce the antiviral IFN pathway^34^. To gain a thorough understanding of the antiviral action of THHY, further investigation of the correlation between THHY and RIG-I/MAVS-mediated antiviral IFN responses is warranted. Moreover, several reports have demonstrated that DENV interrupts and escapes innate host immune responses by interfering IFN mediators. For instance, DENV protease has been indicated to target MAVS^18^, DENV NS4B has been demonstrated to block STAT1 phosphorylation^19^, and DENV NS5 has been reported to block the JAK-STAT2 pathway by degradation of STAT2 protein^15^. Further experiments will be performed to clarify how THHY interrupts the DENV protein-inhibited IFN pathway. In conclusion, our study identified THHY, one of the active constituents of avocado fruit, as a potential agent against DENV infection *in vitro* and *in vivo*. Our results further revealed the detailed mechanism by which THHY suppresses DENV replication via up-regulation of NF-κB–mediated antiviral IFN responses and downstream antiviral gene expression. Additionally, THHY protected ICR suckling mice against death by DENV infection *in vivo*, allowing avocado fruit to serve as a potential dietary resource to develop a supplement for the treatment DENV infection and DENV-related diseases.

## Materials and Methods

### Ethics statement and experimental animals

In this study, the six-day-old ICR suckling mice purchased from BioLasco Taiwan Co Ltd were maintained under specific pathogen-free conditions for the anti-DENV efficacy of THHY. The experimental protocol was approved by the Animal Research Committee of Kaohsiung Medical University of Taiwan (IACUC, 103164) according to the guidance of the Public Health Service (PHS) Policy on Humane Care and Use of Laboratory Animals.

### Chemicals

(2*R*,4*R*)-1,2,4-trihydroxyheptadec-16-yne (THHY) (Fig. 1B) was extracted from the unripe pulp of avocado^27^. Ribavirin was purchased from Sigma Chemical Co. (St. Louis, MO, USA). The compound was dissolved in a 100 mM stock solution of dimethyl sulfoxide (DMSO) and stored at −20° C. In each reaction, the final DMSO concentration was 0.1%.

### Cells and virus

Human hepatoma Huh-7 cells were cultured in Dulbecco’s Modified Eagle’s medium (DMEM) supplemented with 10% fetal bovine serum, 1% nonessential amino acids, and 1% antibiotic–antimycotic at 37 °C. C6/36 mosquito cells were cultured in RPMI 1640 medium supplemented with 10% FBS, 1% nonessential amino acids, 1% L-glutamine, 1% sodium pyruvate, and 1% antibiotic–antimycotic in a 5% CO2 atmosphere at 37 °C. DENV-2 strain 16681 was kindly provided by Dr. Huey-Nan Wu (Institute of Molecular Biology, Academia Sinica, Taipei, Taiwan). The other types of DENV (DENV-1:DN8700828; DENV-3: DN8700829; DENV-4: S9201818) were provided by the Centers for Disease Control, Department of Health, Taiwan. DENV was propagated in mosquito C6/36 cells (Hsu *et al*., 2012; Lee *et al*., 2015). Virus titer was determined by the TCID_50_ method^2^.

### Plasmid

pISRE-Luc harboring IFN-stimulated response element (ISRE)-driven firefly luciferase was used to measure IFN-stimulated transcriptional activity (Stratagene, Agilent Technologies, Palo Alto, CA, USA). pNF-κB-Luc harboring NF-κB binding element-driven firefly luciferase was used to measure NF-κB transcriptional activity (BD Biosciences Clontech, Palo Alto, CA, USA)^35^.

### Quantification of RNA levels

Total cellular RNA was extracted by using an RNA Purification Kit (GMbiolab Co, Ltd., Taichung, Taiwan) following the manufacturer’s instructions. The relative DENV RNA or cellular mRNA levels were measured by quantitative real-time reverse-transcription polymerase chain reaction (qRT-PCR) with specific primers (Table 1) following normalization of cellular glyceraldehyde-3-phosphate dehydrogenase (*gapdh*) mRNA level^20^.

**Table 1:** oligonucleotide sequences for real-time RT-PCR.

Oligonucleotide Name	Sequence 5′-3′
DENV gene oligonucleotide sequences	
Type 1–5′ NS5	5′- CAGGTCAAACGCAGCTATTG
Type 1–3′ NS5	5′- CCACTCCACTGAGTGAATTC
Type 2–5′ NS5	5′- TGTATGCCGATGACACCGCA
Type 2–3′ NS5	5′- TCTTTGCACACGGACCACCT
Type 3–5′ NS5	5′- TCAGAACTAACGCAGCCATG
Type 3–5′ NS5	5′- AGAGTTTTCACGCGAGAACC
Type 4–5′ NS5	5′- AGATCAAACGCAGCCATAGG
Type 4–5′ NS5	5′- CTTCCACTCCACTCCATGAA
**Human gene oligonucleotide sequences**	
5′ GAPDH	5′-GTCTTCACCACCATGGAGAA
3′ GAPDH	5′-ATGGCATGGACTGTGGTCAT
5′ OAS1	5′- CAAGCTTAAGAGCCTCATCC
3′ OAS1	5′- TGGGCTGTGTTGAAATGTGT
5′ OAS2	5′- ACAGCTGAAAGCCTTTTGGA
3′ OAS2	5′- GCATTAAAGGCAGGA AGCAC
5′ OAS3	5′- CACTGACATCCCAGACGATG
3′ OAS3	5′- GATCAGGCTCTTCAGCTTGG
5′ PKR	5′- ATGATGGAAAGCGAACAAGG
3′ PKR	5′- GAGATGATGCCATCCCGTAG
5′ IFN-alpha 2	5′-GCA AGT CAA GCT GCT CTG TG
3′ IFN-alpha 2	5′-GAT GGT TTC AGC CTT TTG GA
5′ IFN-alpha 17	5′-AGG AGT TTG ATG GCA ACC AG
3′ IFN-alpha 17	5′-CAT CAG GGG AGT CTC TTC CA

### Western blotting

The procedure of western blotting was performed as described before^20^. The listed antibodies were used in this study, including anti-DENV NS2B antibody (GeneTex, Inc, Irvine, CA), NF-κB antibody (Cell Signaling Technology, Inc. Beverly, MA), IKK-α antibody (Cell Signaling), IKK-β antibody (Cell Signaling), IκBα antibody (Cell Signaling), STAT1 antibody (GeneTex), STAT2 antibody (GeneTex), phosphorylated NF-κB antibody (Cell Signaling), phosphorylated IKK-α/β antibody (Cell Signaling), phosphorylated IκBα antibody (Cell Signaling), phosphorylated STAT1 (Tyr701) antibody (Cell Signaling), phosphorylated STAT2 (Tyr690) antibody (Cell Signaling), and anti-GAPDH antibody (GeneTex). The GAPDH protein level served as an internal control^20,36^. The DENV nonstructural protein NS2B served as an indicator to represent viral protein synthesis.

### Transfection

Huh-7 cells were seeded in 24-well plates for 24 h, and the cells were transfected with the indicated plasmids by TransIT-X2® transfection reagent (Mirus Bio LLC, Madison, WI, USA) according to the manufacturer’s instructions.

### Measurement of luciferase activity

Luciferase expression was determined by using the Steady-Glo Luciferase Assay System (Promega Corporation, Madison, WI, USA) in accordance with the manufacturer’s instructions.

### Analysis of extracellular IFN-α protein level

Huh-7 cells were infected with DENV at an MOI of 0.2 for 2 h, followed by THHY treatment for 3 days. The supernatant was collected to measure IFN-α concentration by using a human IFN-α ELISA kit (Life Science) in accordance with the manufacturer’s protocol. Absorbance of each condition was detected at 450 nm using an Epoch microplate spectrophotometer (BioTek Instruments Inc, Winooski, VT, USA).

### Anti-DENV activity *in vivo*

The anti-DENV activity *in vivo* was determined as described before^20^. In brief, Six-day-old ICR suckling mice were randomly divided into three groups (n = 5): Group 1 received heat-inactivated DENV and saline treatment (iDENV); Group 2 received DENV and saline treatment (DENV); and Group 3 received DENV and 5 mg/kg of THHY treatment at 1, 3, and 5 days post infection (DENV + THHY 5 mg/kg). The suckling mice were sacrificed at day 6 post-infection by carbon dioxide euthanasia. For determination of virus titer, brain tissues were collected, weighed and homogenized in RPMI 1640 medium. Subsequently, the supernatants were collected and stored at −80 °C after centrifugation at 8000 rpm for 15 min at 4 °C.

### Measurement of viral titers

The determination of viral titer was performed as described before^20^. In brief, Huh-7 cells were infected by serially diluted virus. Following incubation for 2 h, the virus inoculum was removed and replaced with complete growth medium. After 3 days, the DENV-infected cells were fixed with 4% paraformaldehyde for 20 minutes on ice and followed by permeabilization with 0.2% Triton X-100 for 20 minutes on ice. The fixed cells were blocked in 4% BSA for 2 hours, followed by incubation of anti-prM antibody (1:1000, GeneTex) over night. The cells were washed with PBS for 6 times followed by incubation with Alexa Flour 488-conjugated goat anti-rabbit antibody (1:1000, Life Technologies Corporation, Carlsbad, California, USA) for 1 hour. The cells were washed with PBS for 6 times and counterstained with DAPI (1:1000, Life Technologies Corporation, Carlsbad, California, USA) for 5 minutes. The cells were washed with PBS for 6 times and observed with EVOS FL Cell Imaging System (Life Technologies Corporation, Carlsbad, California, USA), and the virus titer was calculated by the TCID_50_ method.

### Statistical analysis

Data are represented as mean ± standard deviations from at least three independent experiments (n ≥ 3). Statistical significance was calculated using Student’s *t* test for differences between 2 data groups (drug-treated and -untreated cells). The experimental values of n indicate the number of experiments used. Results were considered significant at *P < 0.05.

## Electronic supplementary material

Supplementary information
