# Supplementary material for: Avocado (Persea americana) fruit extract (2R,4R)-1,2,4-trihydroxyheptadec-16-yne inhibits dengue virus replication via upregulation of NF-κB–dependent induction of antiviral interferon responses
Source: Sci Rep. 2019 Jan 23;9:423. doi: 10.1038/s41598-018-36714-4 (PMC6344542; doi:10.1038/s41598-018-36714-4)
Supplement: Supplementary file 1 — Supplementary information [file 41598_2018_36714_MOESM1_ESM.pdf]

Avocado (*Persea americana*) fruit extract (2*R*,4*R*)-1,2,4-trihydroxyheptadec-16-yne inhibits dengue virus replication via upregulation of NF-κB–dependent induction of antiviral interferon responses

Yu-Hsuan Wu[1], Chin-Kai Tseng[1], Ho-Cheng Wu[2], Chih-Ku Wei[3], Chun-Kuang Lin[4], Ih-Sheng Chen[5], Hsun-Shuo Chang[5,6*], Jin-Ching Lee[2,3,6,7,8*]

[1]Institute of Basic Medical Sciences, College of Medicine, National Cheng Kung University, Tainan, Taiwan

[2]Graduate Institute of Medicine, College of Medicine, Kaohsiung Medical University, Kaohsiung, Taiwan.

[3]Department of Biotechnology, College of Life Science, Kaohsiung Medical University, Kaohsiung, Taiwan

[4]Doctoral Degree Program in Marine Biotechnology, College of Marine Sciences, National Sun Yat-Sen University, Kaohsiung, Taiwan

[5]School of Pharmacy, College of Pharmacy, Kaohsiung Medical University, Kaohsiung, Taiwan

[6]Graduate Institute of Natural Products, College of Pharmacy, Kaohsiung Medical University, Kaohsiung, Taiwan

[7]Research Center for Natural Products and Drug Development, Kaohsiung Medical University, Kaohsiung, Taiwan.

[8]Department of Medical Research, Kaohsiung Medical University Hospital, Kaohsiung, Taiwan

Running title: Anti-DENV activity of avocado fruit extract

***Corresponding authors:** Jin-Ching Lee and Hsun-Shuo Chang

Mail address: Department of Biotechnology, Kaohsiung Medical University, 100, Shih-Chuan 1st Road, San Ming District, 807 Kaohsiung City, Taiwan.

Phone: 886-7-312-1101 ext. 2369 **Fax:** 886-7-312-5339

E-mail: jclee@kmu.edu.tw and hschang@kmu.edu.tw

Supplementary Figure 1. Full length blot of Figure 1C

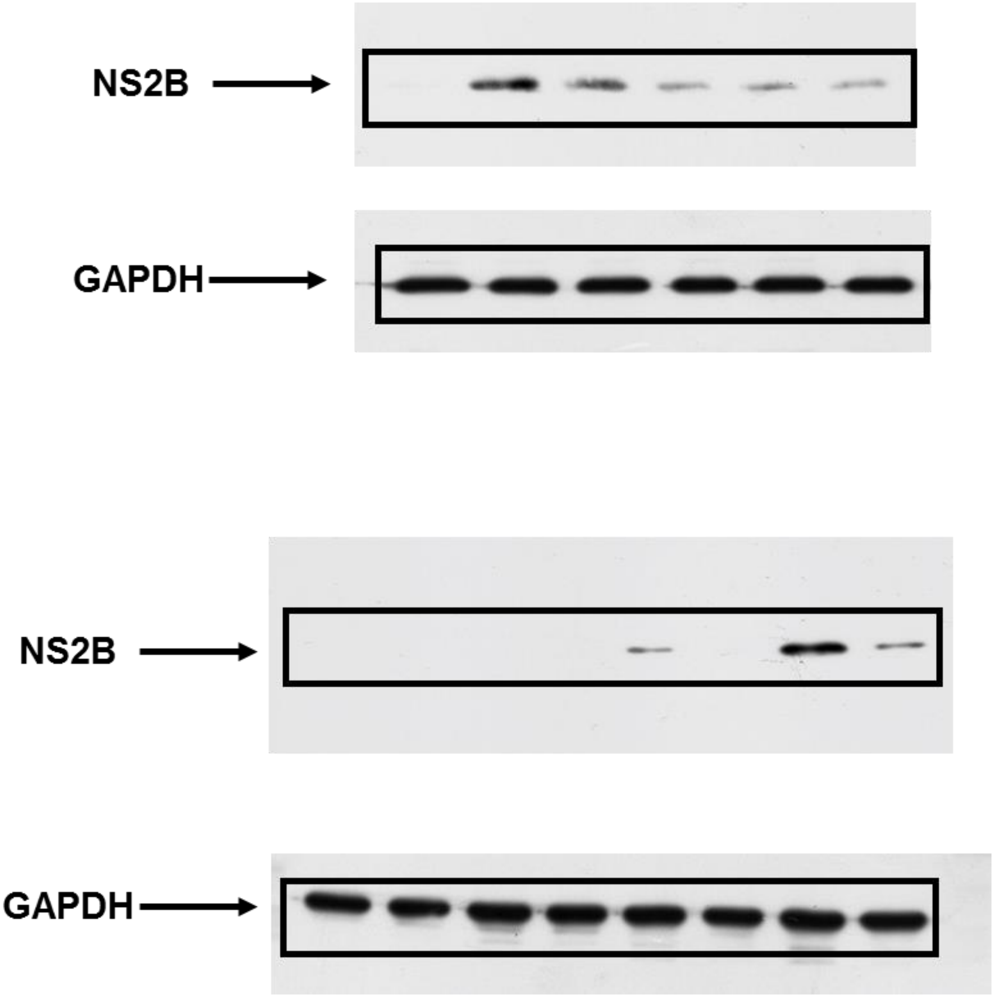

The original full-length immunoblots related to Figure 1C and 1F are shown. The unloaded well indicated that this well did not load protein sample.

Supplementary Figure 2. Full length blot of Figure 2A

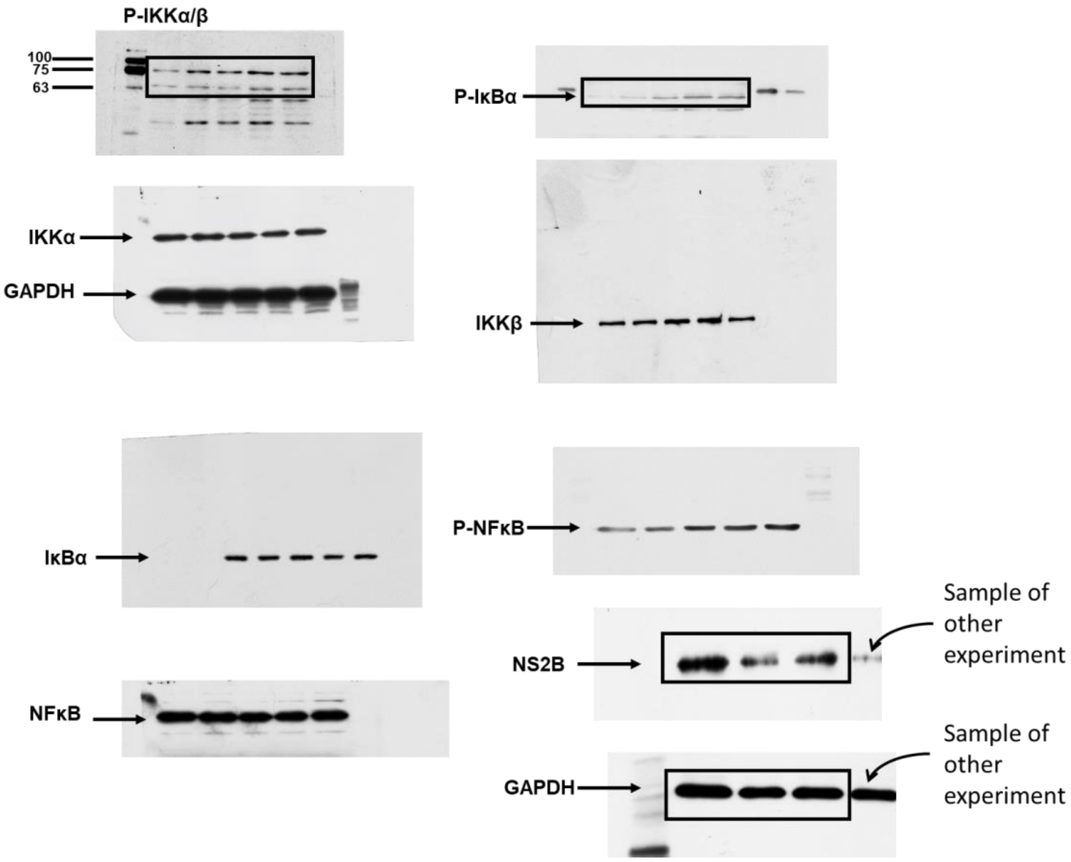

The original full-length immunoblots related to Figure 2A and 2C are shown.

Supplementary Figure 3. Full length blot of Figure 4A

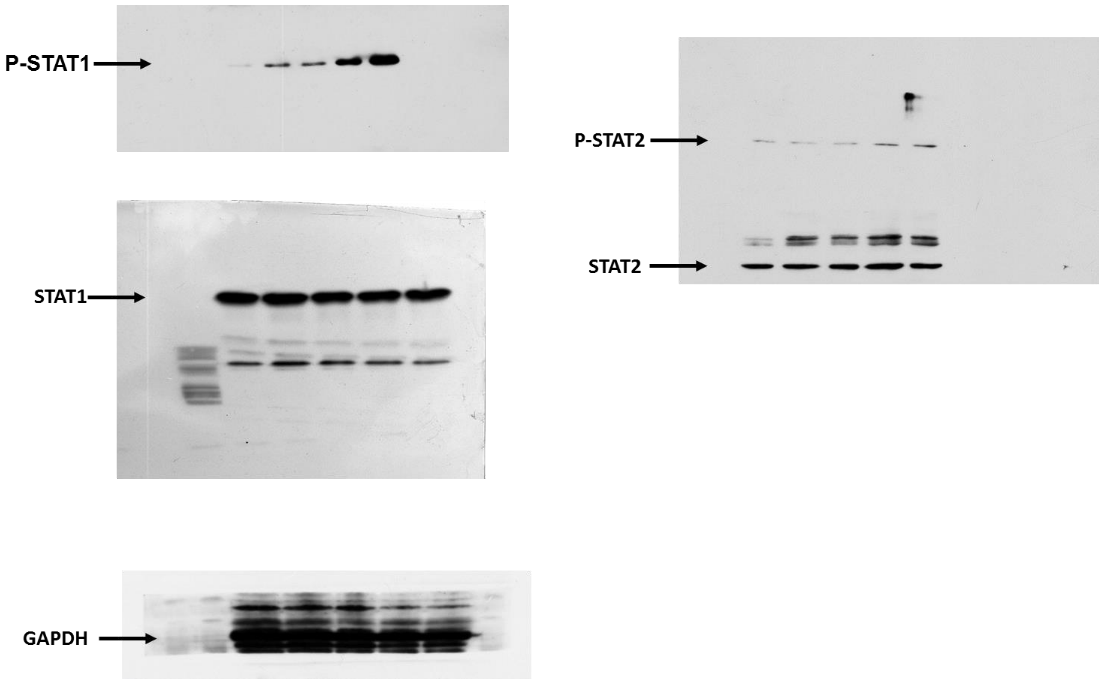

The original full-length immunoblots related to Figure 4A are shown.
